# Long-term outcomes of anti-vascular endothelial growth factor therapy with and without posterior scleral reinforcement on myopic maculopathy in myopic choroidal neovascularization eyes

**DOI:** 10.1186/s12886-024-03357-1

**Published:** 2024-03-13

**Authors:** Meng-Tian Kang, Ningli Wang, Wenjun Xu, Mayinuer Yusufu, Wu Liu, Jiaxin Tian, Yue Qi

**Affiliations:** 1 Beijing Tongren Hospital, Beijing Ophthalmology & Visual Science Key Lab, Beijing Tongren Eye Center, Capital Medical University, Beijing, China; 2 Beijing Institute of Ophthalmology, Beijing Tongren Hospital, Beijing Tongren Eye Center, Capital Medical University, Beijing, China; 3 Centre for Eye Research Australia, Royal Victorian Eye and Ear Hospital, East Melbourne, Australia; 4 Department of Surgery (Ophthalmology), The University of Melbourne, Melbourne, Australia

**Keywords:** Choroidal neovascularization, Myopic maculopathy, Anti-vascular endothelial growth factor, Posterior scleral reinforcement

## Abstract

**Background:**

Anti-vascular endothelial growth factor (anti-VEGF) therapy is used for myopic choroidal neovascularization (mCNV). Patchy chorioretinal atrophy (pCRA) enlargement has been reported in mCNV cases associated with vision loss. Our aim was to compare the long-term effectiveness of anti-VEGF therapy alone versus anti-VEGF followed by posterior scleral reinforcement (PSR) in controlling myopic maculopathy in mCNV eyes.

**Methods:**

We performed a retrospective review of the medical records of 95 high myopia patients (refractive error ≥ 6.00 diopters, axial length ≥ 26.0 mm) with mCNV. Patients were treated with anti-VEGF alone (group A) or anti-VEGF followed by PSR (group B). The following data were collected: refractive error, best corrected visual acuity (BCVA), ophthalmic fundus examination, ocular coherence tomography and ocular biometry at 12 and 24 months pre- and postoperatively. The primary outcomes were changes in pCRA and BCVA.

**Results:**

In 26 eyes of 24 patients, the mean pCRA size significantly increased from baseline (0.88 ± 1.69 mm^2^) to 12 months (1.57 ± 2.32 mm^2^, *t* = 3.249, *P* = 0.003) and 24 months (2.17 ± 2.79 mm^2^, *t* = 3.965, *P* = 0.001) postoperatively. The increase in perilesional pCRA in group B (*n* = 12) was 98.2% and 94.2% smaller than that in group A (*n* = 14) at 12 and 24 months (*Beta* 0.57 [95% CI 0.01, 191 1.13], *P* = 0.048). In group B, 7 eyes (58.3%) gained more than 2 lines of BCVA compared with only 4 eyes (28.6%) in group A at 24 months.

**Conclusion:**

Anti-VEGF therapy followed by PSR achieved better outcomes than anti-VEGF therapy alone in controlling the development of myopic maculopathy in mCNV and may constitute a better treatment option by securing a better long-term VA outcome.

**Supplementary Information:**

The online version contains supplementary material available at 10.1186/s12886-024-03357-1.

## Introduction

Pathologic myopia is the second most common cause of visual impairment in East Asia, with a prevalence of 0.9-3.1% [1–4]. Among all vision loss due to pathologic myopia, 73.3% is caused by myopic maculopathy, including myopic choroidal neovascularization (mCNV) and chorioretinal atrophy [5, 6]. Globally, myopic maculopathy affects approximately 10 million patients, and the associated global potential productivity loss is estimated at US$6 billion [7]. The international photographic classification and grading system classifies myopic maculopathy into five categories, with Category 4, macular atrophy, imposing the most severe vision damage [8]. Considering that mCNV contributes to 92.7% of macular atrophy, effective treatment of mCNV is crucial to avoid progression into macular atrophy to spare patients from vision loss.

The natural course of mCNV can result in expanding macular atrophy, leading to irreversible visual loss after 5 years, and the standard treatment is intravitreal anti-vascular endothelial growth factor (anti-VEGF) therapy. Several studies with 1-year follow-up have shown significant visual acuity improvement after anti-VEGF therapy [9]. However, studies have revealed the long-term outcome of anti-VEGF therapy to be less predictable, and the results are inconsistent [7, 10–12]. A study by Ruiz-Moreno showed that the logarithm of the minimum angle of resolution (logMAR) best-corrected visual acuity (BCVA) averaged 0.54 at baseline, increased to 0.40 at 1 year, and decreased to 0.47 at 2 years, mostly due to the development of patchy chorioretinal atrophy (pCRA) [7]. The incidence of CRA in eyes treated with intravitreal anti-VEGF is 10% at 1 year, 19% at 2 years, and 72% at 6 years in treated eyes [13, 14]. The failure to secure long-term efficacy could be attributable to the fact that anti-VEGF therapy cannot prevent mCNV-related choroidal atrophy. mCNV-related macular atrophy is one of the major contributing factors that undermines the long-term visual prognosis after anti-VEGF treatment in myopic patients [15]. While macular atrophy is identified as the main cause of mCNV-induced vision loss, there is currently no effective treatment for it.

Posterior scleral reinforcement (PSR) is effective in controlling myopia progression by slowing both refraction and axial length(AL) changes [16]. Animal experiments showed that the implanted sclera can repair the sclera through neovascularization [17]. Neovascularization improves the nutritional status of the posterior pole and consequently visual function. Additionally, PSR can increase choroidal perfusion significantly in high myopia patients [18]. Moreover, the stimulation caused by the tearing of the Bruch membrane and the uneven edges of pCRA may also lead to mCNV [19, 20]. The PSR tightens the posterior sclera while integrating with the implant, which consequently restores the broken Bruch membrane and relieves the tension of the edges of pCRA or lacquer cracks, thereby retarding the progression of mCNV-related choroidal atrophy. Patchy atrophy and lacquer cracks predispose patients to the development of choroidal neovascularization in pathological myopia [20]. In addition, in myopic patients, pCRA extends with AL elongation and converges at the macular fovea, thereby leading to macular atrophy.

The current study hypothesized that anti-VEGF therapy followed by PSR can delay macular atrophy and improve the prognosis of mCNV and evaluated the 2-year outcomes of anti-VEGF therapy alone versus anti-VEGF followed by PSR on pCRA progression in high myopia eyes with mCNV.

## Methods

### Study design and patients

We retrospectively reviewed the medical records of 95 patients who were diagnosed with mCNV and treated with anti-VEGF therapy between January 2015 and January 2021 at the ophthalmology department of Beijing Tongren Hospital. Written informed consent was obtained from all patients. Ethics approval was obtained from the institutional review board of Beijing Tongren Hospital (Approval number: TRECKY2019-025).

The inclusion criteria were (1) high myopia, defined by a spherical equivalent (SE) refractive error ≤ -6.00 diopters (D) and AL ≥ 26.0 mm in both eyes, and the difference in AL between the eyes should not be greater than 2 mm; (2) newly diagnosed mCNV; (3) mCNV treated with Ranibizumab/Conbercept 0.5 mg; and (4) at least two years of posttreatment follow-up.

The exclusion criteria were (1) recurrent mCNV; (2) intraocular surgery during the follow-up, such as cataract surgery, vitreoretinal surgery, or glaucoma surgery; (3) coexistence of other ocular disorders, such as glaucoma, diabetic retinopathy, or other retinal vascular diseases; (4) history of vitreoretinal surgery; and (5) history of any treatments for myopic lesions, including laser photocoagulation and photodynamic therapy (PDT) for mCNV.

### Measurements and calculations

The following preoperative information was obtained for analysis: BCVA, retinal fundus photography, fluorescein angiography (FA) (TRC-50X; Topcon Instrument Corp., Tokyo, Japan) or OCT angiography (Optovue Inc., software Version 2015.1.0). Measurement of AL was also recorded (Lenstar LS900; Haag-Streit, Koeniz, Switzerland). Distance BCVA was measured separately for each eye using a logMAR visual acuity (VA) chart (Precision Vision, La Salle, Illinois, USA) at a distance of 5 m. The chart was retroilluminated and had five tumbling “E” optotypes on each line.

Changes in VA after treatment were classified into three categories: improved VA, defined as BCVA improvement of more than 2 lines; stable VA, defined as BCVA improvement of less than 2 lines or unchanged; and decreased VA, defined as BCVA decrease of 1 line or more.

Retinal fundus photography was taken for both eyes at baseline and at 12 and 24 months for longitudinal analysis of the posterior pole, including the optic disc, macular view, and upper and lower temporal arcade. ImageJ software (developed by Wayne Rasband, NIH, Bethesda, MD, USA) was used to assess the extension of pCRA in square millimeters, and all measurements were performed by two masked experienced investigators (WJX and JXT). At baseline, the pCRA area shown on the fundus photograph was outlined. The pCRA areas were measured with fundus photography and divided into two groups: perilesional atrophy (the area around the mCNV) and patchy extralesional atrophy (areas between the temporal vascular arcades). Areas of all pCRA that developed during the 24 months of follow-up were summed for the measurements and considered as one area. The pCRA change around the perilesional and patchy extralesional pCRA was expressed as the difference between the pCRA area at baseline and follow-ups.

### Surgical procedure and anti-VEGF treatment

**Fig. 1:**
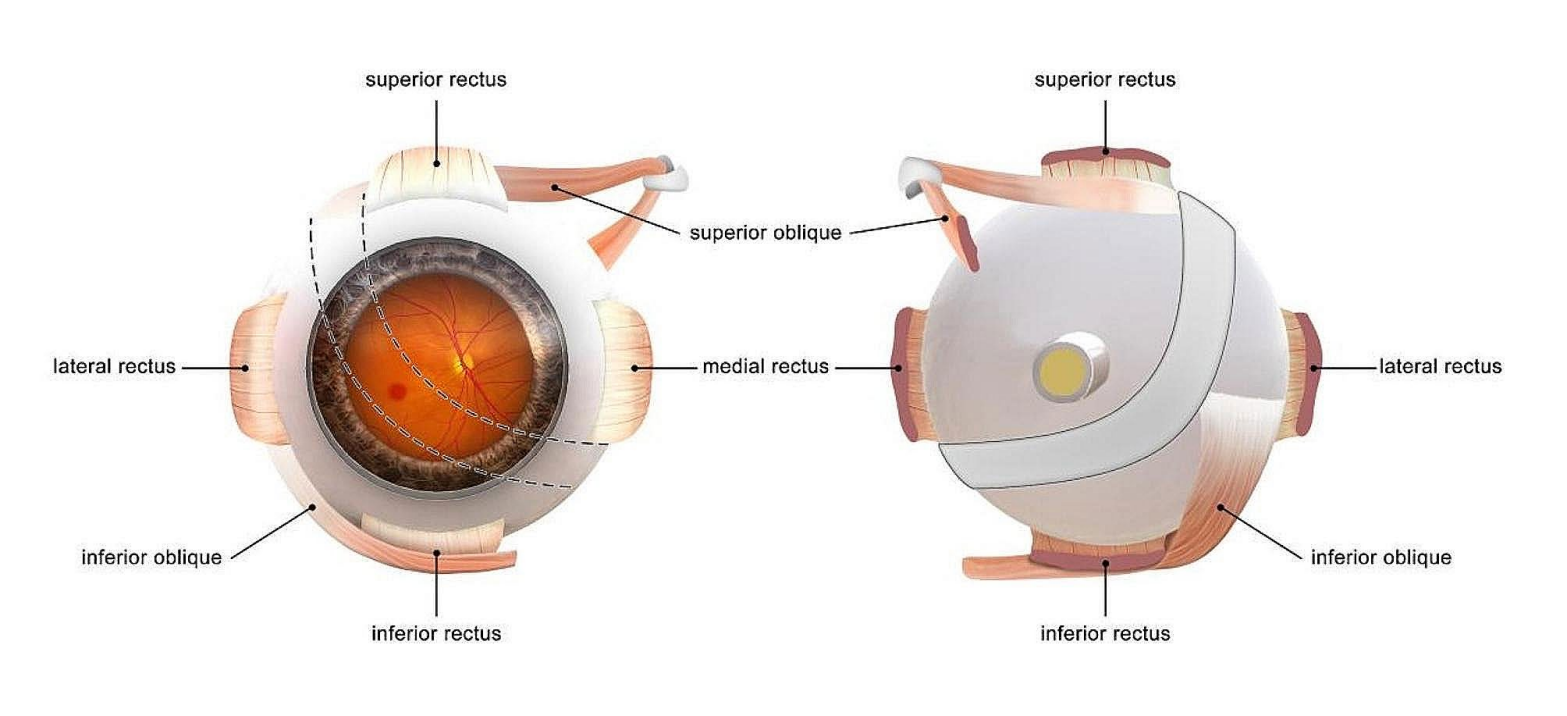
Schematic illustration of the posterior scleral reinforcement surgical procedure

All PSR procedures were performed following the modified Snyder-Thompson procedure (Fig. 1) by the same experienced surgeon (Yue Qi). Under general anesthesia, a lower temporal bulbar conjunctival incision from the edge of the corneal limbus was made, and the lateral rectus muscles, inferior rectus muscles, and inferior oblique muscle were exposed and isolated. A homologous human scleral strip (50 ~ 60 mm long and 6 ~ 10 mm wide) was sequentially inserted under the lateral rectus, inferior oblique, and inferior rectus muscles. One end of the strip was fixed at the temporal side behind the insertion of the superior rectus muscle, while the other end was anchored to the inferior side behind the insertion of the medial rectus muscle with the posterior pole surrounded by the strip. The complications were evaluated during follow-up.

Patients treated with anti-VEGF alone were defined as group A. Patients diagnosed with mCNV were treated with a single intravitreal injection of 0.5 mg/0.05 mL Ranibizumab/Conbercept and followed by a pro re nata (PRN) treatment. The need for retreatment was determined by disease activity. Based on morphologic features that could precede visual loss, the state of active disease was defined as visual impairment due to intra- or subretinal fluid or active leakage secondary to mCNV as seen on OCT or leakage seen on FA [21]. If in the state of active disease, the patient would continue to receive treatment; otherwise, the treatment would be discontinued.

Patients treated with anti-VEGF followed by PSR were defined as group B. The planned time interval between anti-VEGF therapy and PSR was 3 months. After PSR, patients were followed up with PRN treatment.

### Statistical analysis

Data are presented as the mean (standard deviation, SD), median (range) for continuous variables, or percentage together with the confidence interval for categorical variables wherever appropriate. For numerical values, the t test was performed to test the difference between groups for normally distributed data, and the Wilcoxon rank-sum test was used for nonnormally distributed data. For categorical variables, if the expected count was more than 5, the chi-squared test was performed; otherwise, Fisher’s exact test was adopted. To analyze the pCRA progression in different treatment groups, a linear mixed model was performed including baseline pCRA area, time of follow-up, treatment group, and time/treatment interaction terms with the change from baseline of pCRA values as the response variable. The model was estimated using the SAS PROC MIXED procedure with the REML method and unstructured variance‒covariance. The change from baseline of pCRA was log-transformed since it was not normally distributed. The least squares mean method was used to test the difference between groups at different time points. Statistical analyses were performed using the Statistical Analysis System (SAS) software package (version 9.4; SAS Institute Inc., Cary, NC, USA).

## Results

### Baseline

The study included 26 eyes of 24 patients who met the inclusion criteria, with a mean age of 50.9 ± 11.8 years (range 25–74 years). Among them, 12 eyes with previous anti-VEGF injections underwent PSR (group B), and 14 eyes (51.9%) only received anti-VEGF therapy (group A). The mean AL was 30.5 ± 1.0 mm in group B and 30.1 ± 0.5 mm in group A (*F* = 0.743, *P* = 0.402).

Demographic and clinical data are shown in Table 1. During the study period (24 months), patients received an average of 2.5 intravitreal injections (2.5 ± 1.8 [range: 1 to 10]) of Ranibizumab /Conbercept. The average time interval between anti-VEGF therapy and PSR was 3.2 months. In group B, 2 patients had concurrent foveoschisis, and another 2 patients developed mCNV in their fellow eyes. Two eyes in group A had recurrent mCNV during the study period.

The complications of PSR, particularly retinal detachment, optic nerve damage, muscle injury, macular hole, retinal haemorrhage or ischaemia, vortex vein damage, and globe distortion, have been previously reported. In the current study, we did not observe any of these complications during the follow-up period.

**Table 1:** Demographics and clinical data of all eyes and by group

	All	Group A	Group B	*P* value
Number of eyes	26	14	12	
Gender, male/female	8/18 (30.7%)	4/14 (28.6%)	4/12 (33.3%)	1.000
Age, years	51.0 (11.6)	53.9 (11.3)	47.8 (11.6)	0.187
Number of injections	2.5 (1.8)	3.1 (2.2)	1.8 (0.8)	0.075
Baseline logMAR BCVA, letters	0.64 (0.23)	0.64 (0.22)	0.62 (0.25)	0.918
Baseline SE, D	-13.82 (5.24)	-14.01 (5.26)	-13.61 (5.47)	0.853
Baseline AL, mm	30.3 (0.9)	30.1 (0.5)	30.5 (1.0)	0.402
pCRA area, mm^2^	0.88 (1.69)	1.27 (2.14)	0.41 (0.79)	0.201
Perilesional pCRA	0.42 (1.38)	0.69 (1.85)	0.10 (0.33)	0.283
Patchy Extralesional pCRA	0.46 (1.00)	0.58 (1.18)	0.32 (0.77)	0.512
Follow up, months	23.2 (9.6)	22.7 (11.7)	23.9 (6.7)	0.757

*Data are shown as means (standard deviation)

Group A: Patients treated with anti-VEGF alone

Group B: Patients treated with anti-VEGF followed by PSR

### Progression of myopic maculopathy

At baseline, perilesional pCRA was present in 19.2% (5/26) of eyes, and patchy extralesional pCRA at the posterior pole was present in 34.6% (9/26) of eyes.

**Fig. 2:**
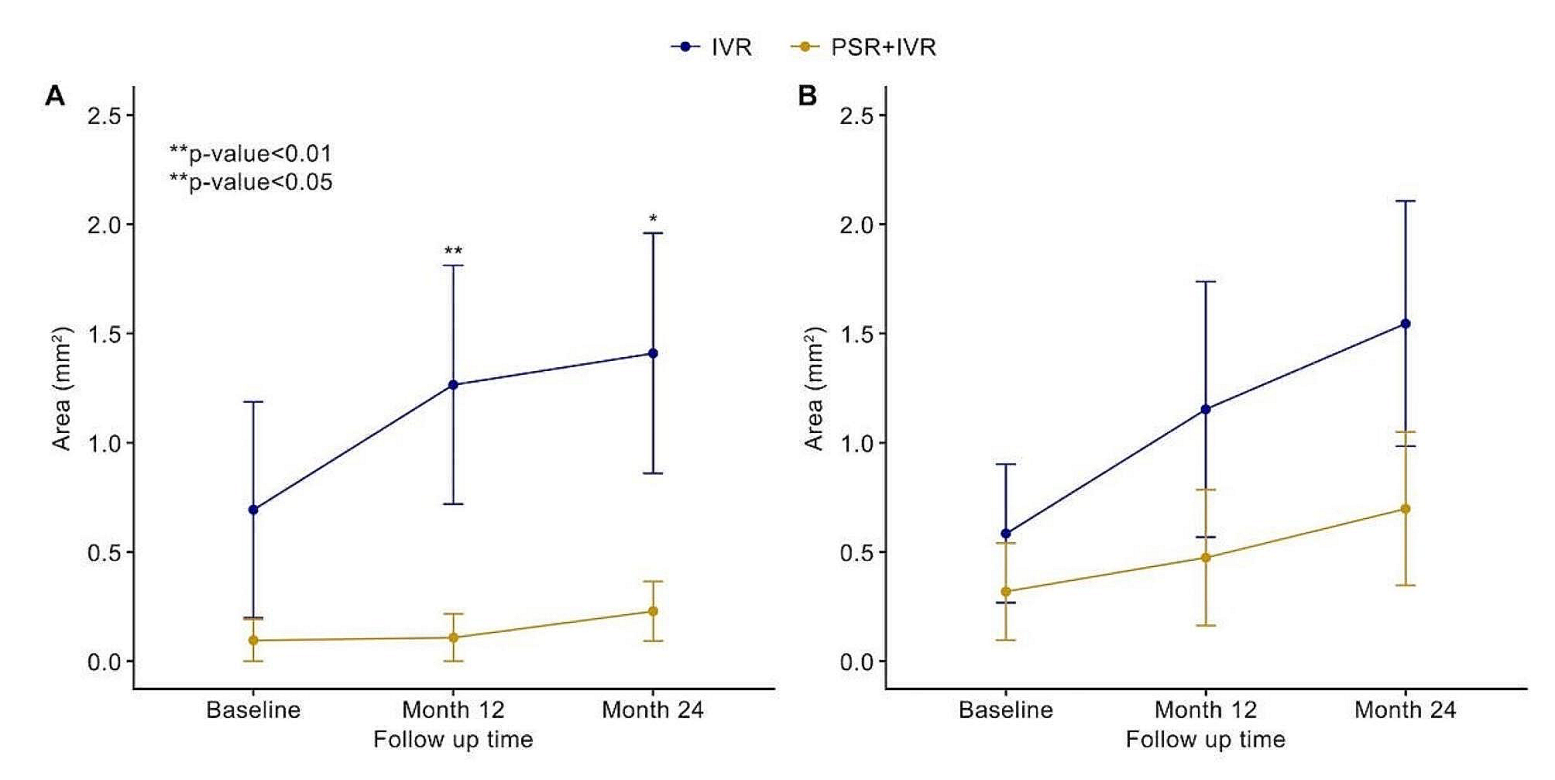
Comparison of CRA progression between group A and group B. Group B (yellow line) showed significantly reduced perilesional CRA progression compared with group A (blue line). (**A**) Perilesional CRA progression after treatment. (**B**) Patchy extralesional CRA progression after treatment. Error bars refer to the standard error of the mean (SEM)

The mean pCRA size significantly increased from baseline (0.88 ± 1.69 mm^2^) to 12 months (1.57 ± 2.32 mm^2^, *t* = 3.249, *P* = 0.003) and 24 months (2.17 ± 2.79 mm^2^, *t* = 3.965, *P* = 0.001) during treatment, and Table 2 shows the progression of perilesional pCRA and patchy extralesional pCRA in group A and group B. The enlargement of perilesional pCRA was 98.2% and 94.2% smaller in group B at 12 months and 24 months compared with group A. The mixed model included the baseline pCRA area, time of follow-up, treatment group, and time/treatment interaction. Larger patchy extralesional pCRA at baseline was associated with greater pCRA progression during follow-up in all patients (Beta 0.70 [95% CI 0.50, 0.89], *P* < 0.0001). The mean change in perilesional pCRA size was not significantly associated with the number of injections, axial length or age (*P* > 0.05). In group A, perilesional pCRA increased significantly compared with group B (Beta 0.57 [95% CI 0.01, 1.13], *P* = 0.048). (Fig. 2)

**Table 2:** Mixed model analysis of pCRA progression with different treatment groups

	Perilesional CRA (mm^2^)			Patchy Extralesional CRA (mm^2^)	
	Beta (95% CI)	*P*		Beta (95% CI)	*P*
Baseline	0.00 (-0.21, 0.20) ^a^	0.9775		0.70 (0.50, 0.89)	**< 0.0001**
Change of 12-month	0.01 (-0.39, 0.41)	0.9589		0.06 (-0.34, 0.22)	0.6864
Change of 24-month	0.14 (-0.28, 0.57)	0.4948		0.26 (-0.10, 0.62)	0.1497
Treatment group					
Group B	Reference			Reference	
Group A	0.57 (0.01, 1.13)	**0.048**		0.21 (-0.17, 0.59)	0.2716

^a^ Estimated beta less than 0.01.

**Fig. 3:**
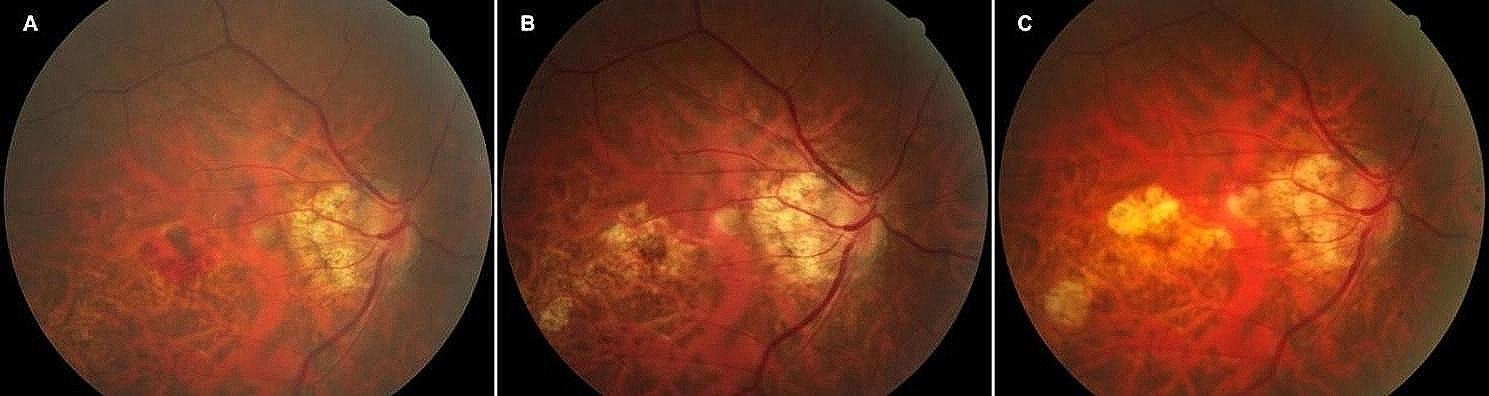
Right fundus of a 61-year-old woman in the anti-VEGF group. Baseline BCVA of 20/2000, -16 D. This patient had a total of 3 anti-VEGF injections and a final BCVA of 20/2000. (**A**) Baseline retinography of CNV diagnosed; (**B**) 12 months after treatment, macular atrophy developed around the regressed CNV; (**C**) 24 months after treatment, patchy atrophy arose around the macula

**Fig. 4:**
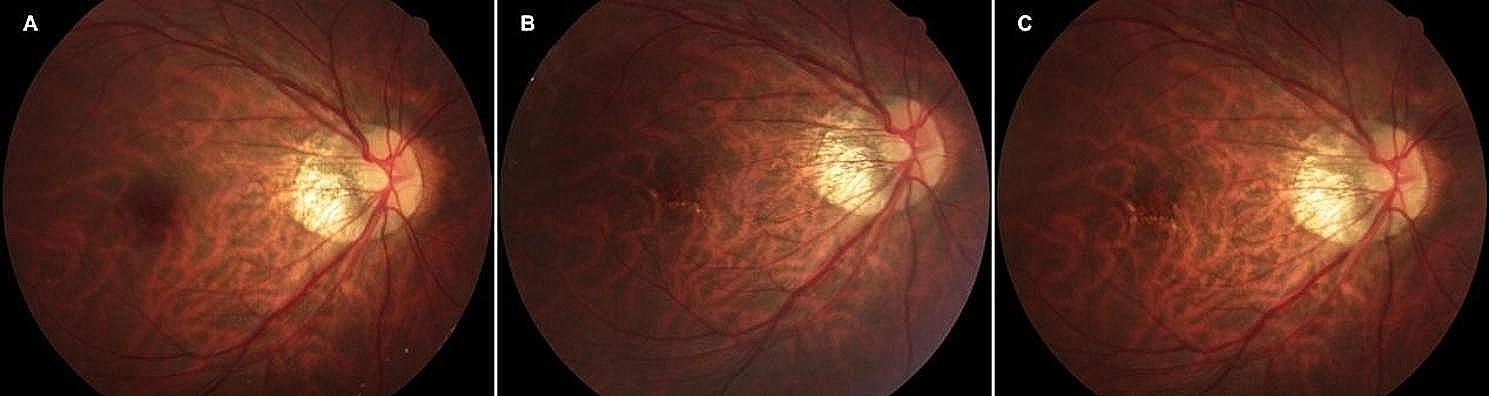
Right fundus of a 40-year-old woman in the anti-VEGF followed by PSR group. Baseline BCVA of 20/200, -6 D. This patient had one injection for anti-VEGF during treatment and a final BCVA of 20/50. (**A**) Baseline retinography of CNV diagnosed, (**B**) 12 months after treatment, (**C**) 24 months after treatment, no patchy atrophy detected

**Fig. 5:**
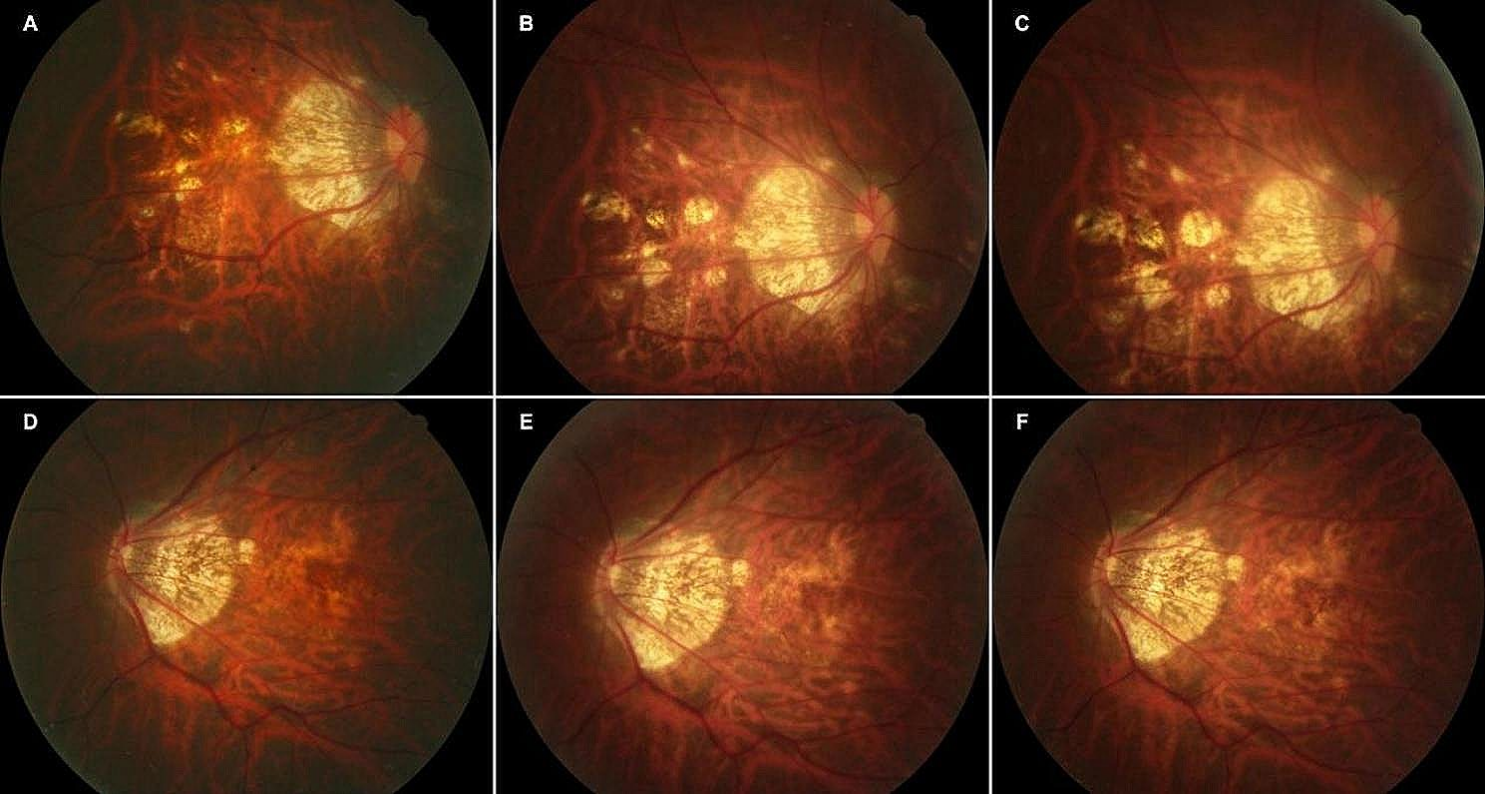
A 54-year-old woman with CNV in both eyes was diagnosed. (**A**) Baseline retinography of CNV diagnosed in the right eye. Baseline BCVA of 20/2000, -16D. (**B**) Twelve months after anti-VEGF treatment, patchy atrophy progression was detected. (**C**) Twenty-four months after anti-VEGF treatment, the area of patchy atrophy increased. A final BCVA of 20/67. (**D**) Baseline retinography of CNV diagnosed in the left eye. Baseline BCVA of 20/67, -17 D. (**E**) Twelve months after anti-VEGF followed by PSR treatment. (**F**) Twenty-four months after treatment, no patchy atrophy was detected around the CNV. A final BCVA of 20/50

Fig. 3, 4 and 5 illustrate the changes in the retinal fundus. The progression of pCRA and the number of intravitreal injections are presented in Supplementary material 1. Group A received an average of 3.07 injections, and group B received an average of 1.83 injections. Myopic schisis involving the fovea was found in only one eye in group B.

### Best corrected visual acuity

**Fig. 6:**
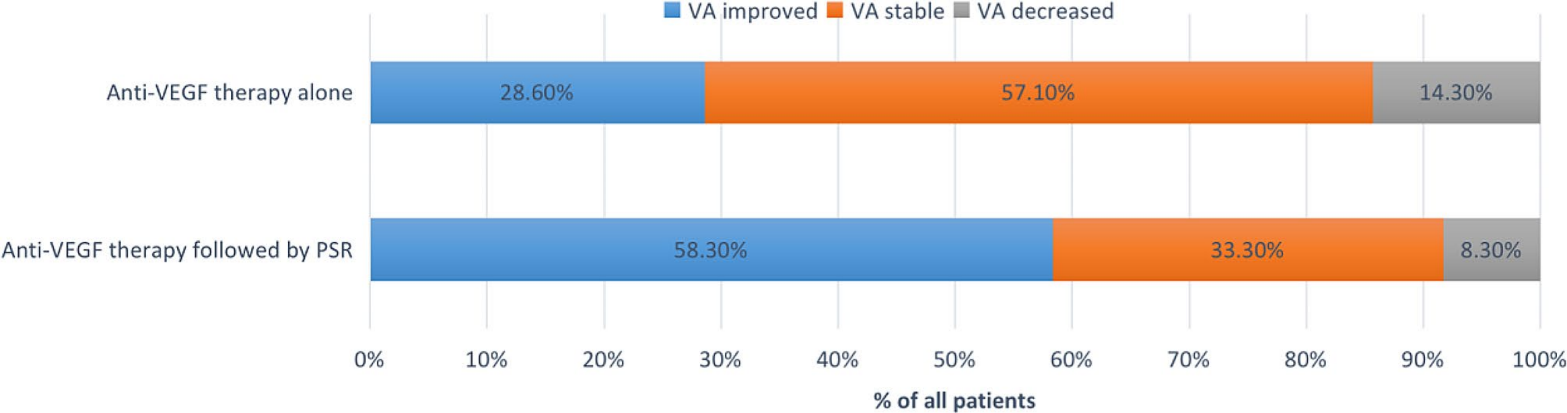
Change in visual acuity (percentage of patients) at 24 months in group A and group B. VA improvement was defined as BCVA improvement of more than 2 lines; VA stability was defined as BCVA improvement of less than 2 lines or unchanged; VA decrease was defined as BCVA decrease of 1 line or more

The baseline logMAR BCVA showed no difference between group A and group B (0.64 ± 0.22 vs. 0.62 ± 0.25, *P* = 0.918). After 24 months, the logMAR BCVA in group B increased by 0.23 ± 0.32 and that in group A increased by 0.09 ± 0.22. (*F* = 0.967, *P* = 0.340) In group A, the logMAR BCVA was improved (VA improvement of more than 2 lines) in 28.6% (4/14) of eyes, remained stable (VA improvement of less than 2 lines or unchanged) in 57.1% (8/14) of eyes, and decreased (VA decrease of 1 line or more) in 14.3% (2/14) of eyes. In group B, the logMAR BCVA was improved in 58.3% (7/12) of eyes and remained stable in 33.3% (4/12) of eyes, while only one (8.3%) out of 12 eyes experienced a VA decrease because of the enlargement of pCRA in the macula area. (Fig. 6)

## Discussion

The current study found that for the treatment of mCNV, anti-VEGF treatment followed by PSR can better delay the expansion of the perilesional CRA area than anti-VEGF treatment alone. The enlargement of perilesional pCRA was 98.2% and 94.2% smaller in anti-VEGF followed by PSR at 12 months and 24 months, respectively, than in anti-VEGF therapy alone. The proportion of patients with improved BCVA was higher in the anti-VEGF group, followed by the PSR group (58.3% vs. 28.6%).

In the current study, 2 patients (1 in group A and 1 in group B) developed binocular mCNV. The patient (No. 18) in group A received anti-VEGF treatment twice in both eyes, and the perilesional CRA area in the right eye enlarged from 0 mm^2^ to 1.254 mm^2^ and that in the left eye from 0 mm^2^ to 0.592 mm^2^. The patient (No. 12) in group B received anti-VEGF treatment followed by PSR in the left eye and anti-VEGF treatment alone in the right eye. The CRA area in the left eye did not progress, but the perilesional CRA area in the right eye expanded from 0.896 mm^2^ to 2.122mm^2^. The difference in the VA outcome indicated that PSR after anti-VEGF treatment could provide additional benefits in controlling the progression of atrophy.

Two patients who received PSR developed concurrent myopic foveoschisis (MF), and no CRA progression was observed by the end of the study. As a common association with high myopia, MF progresses slowly and causes severe central visual loss if untreated. It is believed that the separation of the retinal layers in MF may be due to inward traction caused in part by progressive ectasia of the sclera and the relative resistance to stretching of the inner retinal layers and the retinal vessels. The stiffness of retinal vessels will affect MF development; however, there is no evidence of the association and interaction between MF and CNV.

Most high myopia patients have preexisting anatomical lesions before CNV, including retinal pigment epithelium (RPE) disorder, myopic traction maculopathy, lacquer crack, and choroidal thinning, and are at a higher risk of developing chorioretinal atrophy in the lesion area. The vascular proliferation of CNV causes exudation and hemorrhage. In most cases, the blood vessels are surrounded by proliferated pigment cells, and the CNV contracts and pulls the surrounding single-layer RPE toward the center, thereby forming a ring without RPE around CNV, which eventually leads to apoptosis of normal cells and macular atrophy. Hampton [22] found that the VA of 60% of mCNV patients was ≤ 0.1. The VA of the affected eyes decreased rapidly in the initial stage, and the dropping rate slowed down in the later stage, eventually resulting in atrophy. Tabandeh [23] investigated mCNV patients over 50 years old and found that the typical mCNV lesions were not large, but the VA of a large proportion of patients decreased to 0.1. A 10-year retrospective study [24] on 25 mCNV patients found a VA of ≤ 0.1 in almost all patients. Therefore, it is very important to find a more effective treatment that eliminates mCNV and retards macular atrophy progression.

At present, commonly adopted treatment protocols include anti-VEGF treatment, laser treatment, PDT, and surgical treatment (macular translocation, choroidal neovascularization resection). However, the long-term follow-up of patients treated with anti-VEGF treatment or laser treatment showed that over time, atrophy occurred and progressed in most patients, eventually involving the macular area and resulting in vision loss [25, 26]. PDT intervention could not change the visual outcome either, and those cases always progress into atrophy [27, 28]. While photocoagulation can destroy neovascularization, it also causes RPE injury. PDT also causes choroidal injury and accelerates pathologic myopia-associated atrophy and degeneration. Therefore, the formation of CNV in high myopia and any damage associated with treatment may jointly accelerate this established process. The study found that eyes treated with anti-VEGF may also develop atrophy and reduction of retinal pigment epithelium, with the lesion usually located at the outer boundary, which may be explained by the drug-induced scarring and contraction of CNV lesions, and the contraction would result in annular tear of the retinal pigment epithelium. After years of follow-up, CNV patients developed severe choroidal atrophy and then progressed to chorioretinal atrophy.

Recently, increasing evidence on the association between high myopia and choroidal ischemia has been found. Wu [29] reported on the crucial role of scleral hypoxia in scleral remodeling in the progression of myopia. The study by Zhou [30] showed that eyeball elongation was slowed by choroidal blood perfusion improvement. In addition, a previous study found that PSR slows myopia progression and significantly increases choroidal thickness and choroidal blood flow in high myopia patients. According to animal experiments [17], the histopathological changes after posterior scleral reinforcement were divided into 4 phases: inflammatory reaction period, granuloma formation stage, angiogenesis stage, collagen fiber-formation stage and connective tissue proliferative stage. The implanted sclera began the process of sclera repair via neovascularization 1 to 3 months after the surgery. Neovascularization improves the nutritional status of the posterior pole of high myopia, thereby improving the visual function of the patient. Additionally, during the reconstruction of the implant material, ocular AL can be slightly shortened because of the pulling of the collagen fibers. PSR can delay RPE mechanical damage caused by high myopia by mechanically reinforcing the sclera. Therefore, it is speculated that strengthening scleral collagen fibers by PSR, reducing RPE mechanical injury and increasing choroidal blood flow can decelerate macular atrophy to realize the goal of preserving macular vision. In this study, patients were treated with PRN regiments for 2 years. The average number of injections in group B was lower compared with group A (1.8 ± 0.8 vs. 3.1 ± 2.2, *P* = 0.075), which also testifies to its therapeutic effect as it requires lower injection frequency. In addition, the need for repeated injections can impose an economic burden on the patients.

This two-year retrospective study showed that in patients who received PSR after anti-VEGF treatment, the enlargement of perilesional pCRA at 12 months and 24 months was 98.2% and 94.2% smaller compared with those receiving anti-VEGF treatment alone. Therefore, PSR after anti-VEGF treatment has the potential to act as a booster to further consolidate the treatment efficacy and secure a relatively better 2-year VA outcome. It is worth noting that larger patchy extralesional perilesional CRA at baseline was associated with greater perilesional CRA progression during follow-up in all patients (Beta 0.70 [95% CI 0.50, 0.89], *P* < 0.0001). This suggests the importance of treating mCNV as early as possible before macular atrophy occurs. Even though no statistical difference was found in baseline perilesional CRA areas between groups, there is a 7-fold difference in the mean CRA between group A (0.69) and group B (0.10). This variance may be attributed to the wide variability within group A (1.85) in comparison to group B (0.33). This disparity is primarily influenced by patient No. 15. Approximately 200 million people around the world are affected by pathologic myopia, and in East Asia alone, 11 to 25 million pathologic myopia patients (with an average age of 51) suffer from visual impairment, with approximately 2 to 5 million resulting from pCRA [1, 6, 31, 32]. Million patients could benefit from PSR following anti-VEGF therapy, thereby preserving their VA and improving their quality of life. In particular, considering the increasing prevalence of myopia and high myopia worldwide and that most patients are of working age, a better long-term VA outcome would play a significant role in securing stable income and reducing the economic burden on their families.

There are a few limitations to the current study. First, due to the exploratory inherent limitations of the retrospective study, the patients included in the analysis may have received two different anti-VEGF drugs (Ranibizumab and Conbercept), which may constitute a confounding factor, as the therapeutic effects of different drugs would differ. The time of PSR after anti-VEGF therapy may differ among patients. In the current study, the number of intravitreal injections did not influence pCRA changes over time, even in eyes with mCNV. However, since a PRN regimen was used, this conclusion cannot be extended to other regimens of treatment, where the dose–response effect could be higher. In addition, the small sample size may undermine the statistical power. In previous studies on anti-VEGF treatment for mCNV, vision loss usually occurred 30 months after treatment, and the follow-up period of the current study was only 24 months. After 24 months, the rate of loss to follow-up was relatively high, and thus, it was difficult to carry out further analysis. Therefore, the current study was not able to observe the longer-term VA change. Future prospective, longitudinal studies with a longer period of follow-up are needed to confirm the findings of the current study. Furthermore, as the increase in the choroidal atrophy area is related to AL growth, analysis of the AL changes should also be included in future studies. Several studies have revealed that the severity of choroidal atrophy is associated with older age, more myopic refractive error, longer axial length, staphyloma and worse visual acuity [5, 33, 34]. Our study included patients with an age range spanning from 25 to 74 years and a refractive error range of -6D to -17D. While the difference between the two groups was not statistically significant, the large scale would affect the homogeneity of the statistical analysis.

In conclusion, PSR could retard the progression of mCNV-related choroidal atrophy in highly myopic patients, thereby preserving their VA and improving their quality of life. Therefore, anti-VEGF followed by PSR may constitute a better treatment option for pathological myopic patients with mCNV by securing a better long-term VA outcome.

### Electronic supplementary material

Below is the link to the electronic supplementary material.

Supplementary Material 1

Supplementary Material 2

## Data Availability

The datasets used and analysed during the current study are available from the corresponding author on reasonable request.
