# Supplementary material for: Long-term outcomes of anti-vascular endothelial growth factor therapy with and without posterior scleral reinforcement on myopic maculopathy in myopic choroidal neovascularization eyes
Source: BMC Ophthalmol. 2024 Mar 13;24:118. doi: 10.1186/s12886-024-03357-1 (PMC10938773; doi:10.1186/s12886-024-03357-1)
Supplement: Supplementary file 1 — Supplementary Material 1 [file 12886_2024_3357_MOESM1_ESM.docx]

| **Supplementary material 1. Progression of pCRA and number of intravitreal injections in the treated eyes at baseline, 12 and 24 months** | | | | | | | | |
| --- | --- | --- | --- | --- | --- | --- | --- | --- |
| Pt No | Treated eye | Perilesional pCRA baseline, mm^2^ | Perilesional pCRA 12 m, mm^2^ | Perilesional pCRA 24 m, mm^2^ | Patchy Extralesional pCRA baseline, mm^2^ | Patchy Extralesional pCRA 12 m, mm^2^ | Patchy Extralesional pCRA 24 m, mm^2^ | anti-VEGF total |
| 1 | L | 0 | 0 | 0 | 0 | 0 | 0 | 3 |
| 2 | R | 0 | 0 | 0 | 0 | 0 | 0 | 3 |
| 3 | L | 1.151 | 1.295 | 1.609 | 0 | 0 | 0 | 2 |
| 4^a^ | R | 0 | 0 | 0 | 0 | 0 | 0 | 1 |
| 5 | R | 0 | 0 | 0 | 0 | 0 | 0 | 3 |
| 6 | L | 0 | 0 | 0.126 | 2.474 | 2.861 | 3.023 | 1 |
| 7^b^ | R | 0 | 0 | 0.046 | 1.243 | 2.67 | 3.399 | 2 |
| 8 | L | 0 | 0 | 0 | 0 | 0 | 0 | 1 |
| 9 | L | 0 | 0 | 0 | 0.093 | 0.141 | 0.425 | 2 |
| 10 | R | 0 | 0 | 0 | 0 | 0 | 0 | 1 |
| 11 | R | 0 | 0 | 0 | 0 | 0 | 0 | 1 |
| 12^△^ | L | 0 | 0 | 0 | 0 | 0 | 0 | 2 |
| 12 | R | 0.896 | 1.844 | 2.122 | 0.838 | 1.89 | 2.653 | 2 |
| 13 | L | 1.976 | 2.143 | 2.895 | 1.671 | 2.419 | 3.012 | 3 |
| 14 | R | 0 | 0 | 0.105 | 0 | 0 | 0 | 2 |
| 15 | L | 6.806 | 7.436 | 8.233 | 0 | 0 | 0.132 | 2 |
| 16 | R | 0.068 | 1.244 | 2.119 | 0 | 0 | 0.32 | 5 |
| 17 | R | 0 | 0 | 0.31 | 0 | 0 | 2.171 | 10 |
| 18 | R | 0 | 0 | 1.254 | 4.319 | 8.274 | 8.922 | 2 |
| 18 | L | 0 | 0.229 | 0.592 | 0.225 | 0.609 | 0.81 | 2 |
| 19 | R | 0 | 1.791 | 2.677 | 0.28 | 0.381 | 0.689 | 3 |
| 20 | R | 0 | 2.945 | 3.867 | 0 | 0.535 | 1.213 | 3 |
| 21 | L | 0 | 1.025 | 1.226 | 0.826 | 0.84 | 0.921 | 2 |
| 22 | L | 0 | 0.057 | 0.091 | 0 | 0.153 | 0.254 | 2 |
| 23 | R | 0 | 0 | 0 | 0 | 0 | 0 | 2 |
| 24 | R | 0 | 0.21 | 1.339 | 0 | 0.997 | 1.858 | 3 |
| a. Patient had concurrent foveoschisis.  b. Patient No. 7 was diagnosed with mCNV 5 months after receiving PSR treatment. | | | | | | | | |
