# Supplementary figures and images for: Long-term outcomes of anti-vascular endothelial growth factor therapy with and without posterior scleral reinforcement on myopic maculopathy in myopic choroidal neovascularization eyes
Source: BMC Ophthalmol. 2024 Mar 13;24:118. doi: 10.1186/s12886-024-03357-1 (PMC10938773; doi:10.1186/s12886-024-03357-1)

Supplementary material 2. Study flow chart.


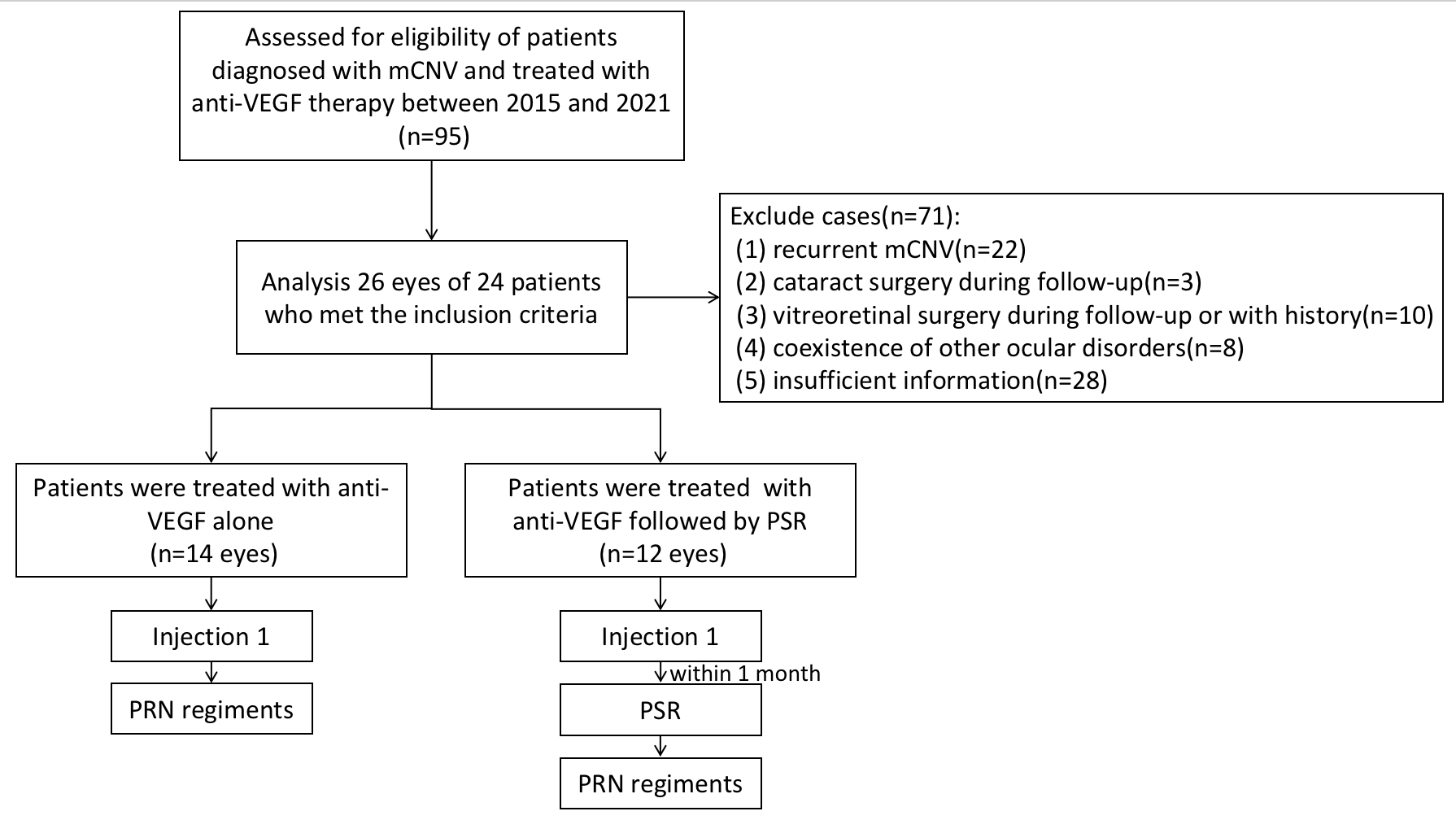

Supplement: Supplementary file 2 — Supplementary Material 2 [file 12886_2024_3357_MOESM2_ESM.docx]
